# Comparison of capecitabine and 5-fluorouracil in chemoradiotherapy for locally advanced pancreatic cancer

**DOI:** 10.1186/1748-717X-8-160

**Published:** 2013-07-03

**Authors:** Yeon-Joo Kim, Woo Jin Lee, Sang Myung Woo, Tae Hyun Kim, Sung-Sik Han, Bo Hyun Kim, Sung Ho Moon, Sang Soo Kim, Young Hwan Koh, Sang-Jae Park, Joo-Young Kim, Dae Yong Kim, Joong-Won Park

**Affiliations:** 1 Center for Liver Cancer, Research Institute and Hospital, National Cancer Center, Goyang, Republic of Korea

**Keywords:** Pancreatic cancer, Chemoradiotherapy, 5-Fluorouracil, Capecitabine

## Abstract

**Background:**

Although capecitabine has theoretical advantages in the pharmacokinetics, such as higher intratumoral and lower systemic concentration, relative to bolus 5-fluorouracil (5-FU), outcomes of chemoradiotherapy (CRT) with capecitabine or bolus 5-FU have not been directly compared in patients with locally advanced pancreatic cancer. Therefore, we retrospectively compared the outcomes, including toxicity, tumor response, and overall survival, of oral capecitabine plus radiotherapy (RT) with bolus 5-FU plus RT, in patients with locally advanced pancreatic cancer.

**Methods:**

Between August 2006 and January 2012, 98 patients with locally advanced pancreatic cancer received CRT, with 52 receiving concurrent oral capecitabine and 46 receiving bolus injection of 5-FU. Primary tumor and overall response after CRT were evaluated radiologically, and toxicity, tumor response, and overall survival (OS) were compared in the two groups.

**Results:**

Baseline clinical parameters of the two groups were similar. The rates of ≥ Grade 3 hematologic (0% vs. 8.7%, *p* = 0.045) and non-hematologic (0% vs. 8.7%, *p* = 0.045) toxicities were significantly lower in the capecitabine group than in the 5-FU group. Primary tumor (30.7% vs. 28.2%, *p* = 0.658) and overall (13.7% vs. 15.2%, *p* = 0.273) response rates and median OS time (12.5 months vs. 11.6 months, *p* = 0.655) were similar in the two groups.

**Conclusions:**

Capecitabine plus RT may be a safe and feasible regimen for patients with locally advanced pancreatic cancer, with similar efficacy and low rates of toxicities compared with bolus 5-FU plus RT.

## Introduction

Surgical resection is the only curative treatment for pancreatic cancer, but only 10–15% of patients have localized and resectable disease at diagnosis. Approximately 50% of pancreatic cancer patients present with distant metastatic disease and 30% present with localized and unresectable disease. The Gastrointestinal Tumor Study Group (GITSG) trials [1-3] showed that chemoradiotherapy (CRT) with bolus injection of 5-fluorouracil (5-FU) yielded a modest survival benefit when compared with radiotherapy (RT) or chemotherapy alone. Since then, CRT plus bolus 5-FU has been regarded as a standard therapy for patients with locally advanced pancreatic cancer. Despite recent advances in diagnostics and therapeutics, the prognosis of patients with locally advanced pancreatic cancer has remained poor, due to high rates of local progression and distant metastasis. Thus, various chemotherapeutic regimens, with various dosages and schedules and with or without RT, have been tested to improve survival [4-8].

Capecitabine, an oral prodrug of 5-FU, is absorbed by the gastrointestinal tract and metabolized to 5-FU by a cascade of three enzymes. Capecitabine is converted by carboxylesterase in the liver to 5′-deoxy-5-fluorocytidine (5′-DFCR), by cytidine deaminase in the liver and tumor tissue to 5′-deoxy-5-fluorouridine (5′-DFUR), and by thymidine phosphorylase (TP) to 5-FU in tumor tissue. TP is more concentrated in tumor tissue than in normal tissue, and is upregulated by radiation in tumor tissue but not in normal tissue. Thus, oral capecitabine can result in a higher intratumoral and lower systemic 5-FU concentration than bolus 5-FU [9,10]. This improved therapeutic index, along with more favorable pharmacokinetics (similar to those of protracted infusion of 5-FU), and convenient oral administration without the need for central venous access and an ambulatory infusion pump, make capecitabine particularly appealing to use in conjunction with RT. To our knowledge, however, outcomes of CRT with capecitabine or bolus 5-FU have not been directly compared in patients with locally advanced pancreatic cancer. We therefore retrospectively compared the outcomes, including toxicity, tumor response, and overall survival, of oral capecitabine plus RT with bolus 5-FU plus RT, in patients with locally advanced pancreatic cancer.

## Methods

### Patients

Between August 2006 and January 2012, 52 patients with primary locally advanced pancreatic cancer underwent CRT with capecitabine under a phase II protocol. Eligibility criteria included: *(1)* pathologically confirmed pancreatic carcinoma; *(2)* unresectable disease (stage cT4) on computed tomography (CT)/positron-emission tomography (PET); *(3)* radiographically assessable disease; *(4)* age ≥ 18 years; *(5)* performance status of 0 to 1 on the Eastern Cooperative Oncology Group (ECOG) score; *(6)* adequate bone marrow (white blood cell count ≥ 2,000/mm^3^, hemoglobin ≥ 7.5 g/dL, platelet count ≥ 100,000/mm^3^), liver (total bilirubin ≤ 3.0 mg/dL), and renal (serum creatinine ≤ 1.5 mg/dL) function; and *(7)*oral intake (including J-tube feeding) of ≥ 1,500 calories/day. Patients with elevated bilirubin due to obstruction were stented and their bilirubin decreased to ≤ 3.0 mg/dL, and patients with biliary or gastroduodenal obstruction underwent drainage prior to study entry. Exclusion criteria included: *(1)* radiographic evidence of metastasis in the major viscera or peritoneal seeding on CT and/or PET; *(2)* previous history of RT adjacent to the planned field; *(3)* pregnancy or breast feeding; and *(4)* previous history of uncontrolled other malignancies within 2 years. All patients provided written informed consent before study enrollment and this trial was registered at clinicaltrials.gov (NCT00658840).

During the same study period, 46 patients who refused to participate in this protocol received CRT plus 5-FU, the routine clinical practice regimen in our institution for patients with locally advanced pancreatic cancer. This study was conducted in accordance with the guidelines of the institutional review boards of the National Cancer Center.

Before CRT, patients were given physical examinations and underwent blood tests, including complete blood count, liver function tests, and serum CA 19–9 concentrations; chest radiography, and dynamic CT and/or PET of the abdomen and pelvis. All tumors were staged using the American Joint Committee on Cancer (AJCC), 6^th^ edition, and were classified as stage cT4 (unresectable disease), based on the CT scans, with tumor extension to the celiac axis or superior mesenteric artery or occlusion of the superior mesenteric-portal venous confluence. Primary tumors were measured bi-dimensionally, with lymph node involvement defined by the presence of a lymph node ≥1 cm in the short-axis, with a spiculated or indistinct border, or with a mottled heterogeneous pattern on CT with or without PET (n = 87) [11]. Table 1 shows the baseline patient characteristics.

**Table 1:** Patient characteristics

**Characteristic**		**Capecitabine (n = 52), n (%)**	**5-FU (n = 46), n (%)**	***p-*value**
Gender	Male	32 (61.5)	31(67.4)	0.173^†^
	Female	20 (38.5)	15 (32.6)	
Age (years)	Median (range)	63 (36–77)	66 (41–80)	0.126^‡^
	Mean ± SE	62.1 ± 1.3	65.0 ± 1.4	
	<65	31 (59.6)	19 (41.3)	0.105^†^
	≥65	21 (40.4)	27 (58.7)	
Tumor location	Head	30 (57.7)	30 (65.2)	0.445^†^
	Body/tail	22 (42.3)	16 (34.8)	
Tumor size^*^ (cm)	Median (range)	3.8 (2.4–7.4)	3.9 (2.5–10)	0.297^‡^
	Mean ± SE	4.0 ± 0.1	4.3 ± 0.2	
	<4	28 (53.8)	23 (50)	0.840^‡^
	≥4	24 (46.2)	23 (50)	
Histological differentiation	Well/moderate	13 (25)	12 (26.1)	0.617^†^
	Poor	2 (3.9)	4 (8.7)	
	Not specified	37 (71.1)	20 (65.2)	
cN classification	N0	32 (61.5)	25 (54.3)	0.471^†^
	N1	20 (38.5)	21 (45.7)	
CEA (ng/ mL)	Median (range)	4.2 (1.2–31.9)	3.8 (1.0–56.1)	0.493^‡^
	Mean ± SE	6.4 ± 0.9	7.7 ± 1.7	
	<5	31 (59.6)	29 (63.0)	0.836^†^
	≥5	21 (40.6)	17 (37.0)	
Pretreatment CA 19–9 level (U/mL)	Median (range)	218.5 (5.0–11445)	191.5 (5.0–4150)	0.056^‡^
	Mean ± SE	1179.6 ± 307.3	531.1 ± 130.6	
	<400	29 (55.8)	31 (67.4)	0.300^†^
	≥400	23 (44.2)	15 (32.6)	

Abbreviations: *FU* fluorouracil, *SE* standard error, *CEA* carcinoembryonic antigen, *CA* 19–9 carbohydrate antigen 19–9, *CRT* chemoradiotherapy.

^*^Maximum diameter of the primary tumor.

^†^Fisher’s exact test, two-tailed.

^‡^*t*-test, two-tailed.

### Treatment

#### *Radiotherapy*

Prior to RT, all patients underwent CT simulation, with their targets defined in accordance with the International Commission on Radiation Units and Measurements Report 50. The gross tumor volume (GTV) encompassed the gross tumor, as defined by contrast CT or PET scan. The clinical target volume (CTV) included the GTV and the volumes of regional lymph nodes, including the porta hepatic, pericholedochal, celiac, and pancreaticoduodenal nodes. The initial and boot planning target volume (PTV) included the CTV and GTV plus a 5–10 mm margin. All patients underwent three-dimensional treatment planning, such that the PTV would be encompassed by a 90% isodose volume of the prescribed dose.

An initial dose of 45 Gy in 25 fractions was delivered to the primary tumor and regional lymph nodes, followed by a boost of 10.8 Gy in 6 fractions to the gross tumor, 5 days a week. All patients received a total radiation dose of 55.8 Gy in 31 fractions.

#### *Chemotherapy*

Capecitabine or 5-FU was delivered concurrently with RT. The capecitabine group received an oral dose of 800 mg/m^2^ twice daily for the duration of RT with weekend breaks. The 5-FU group received two cycles of intravenous bolus injection of 5-FU (400 mg/m^2^/d) for 3 days in the first and fifth weeks of RT.

#### *Treatment after CRT and evaluation*

Following completion of CRT, patients were evaluated clinically and underwent CT scans of the abdomen, chest radiography, and serum CA 19–9 measurements to determine tumor response and resectability. Patients who had resectable disease after CRT was considered for surgical resection, whereas patients who still had unresectable disease were considered for gemcitabine based chemotherapy (1000 mg/m^2^over 30 minutes intravenously once weekly for 3 of every 4 weeks). Patients who refused further chemotherapy or had poor performance status received supportive care.

Serum samples for measurement of CA 19–9 were obtained from all patients within 2 weeks of the initiation of CRT (pre-treatment level) and 1 month after CRT (post-treatment level). The percent decrease in CA 19–9 concentration was calculated as: CA 19–9 percent decrease (%) = [(pre-treatment CA 19–9 – post-treatment CA 19–9)/pre-treatment CA 19–9] × 100. Tumor responses were determined by comparison of CT scans taken before and 1 month after CRT using the Response Evaluation Criteria in Solid Tumors guidelines [12]. A complete response (CR) was defined as the disappearance of the primary tumor. A partial response (PR) was defined as ≥30% reduction in the longest diameter of the primary tumor. Progressive disease (PD) was defined as a ≥20% increase in the longest diameter of the primary tumor or the appearance of one or more new lesions. Stable disease (SD) was defined as a response that did not qualify as a PR or a PD. Objective response rates were calculated as the rate of CR + PR. Patients with CR or PR were considered “Responders”, and those with SD or PD were considered “Non-responders”. Toxicity was recorded according to the National Cancer Institute Common Terminology Criteria for Adverse Events, version 3.0. Due to difficulties in accurately scoring lower grades of acute toxicity in patients with pancreatic cancer, only grade 3 or higher acute toxicities are compared.

### Follow-up and statistical analysis

After completion of treatment, patients were given follow-up examinations every 2–4 weeks for the first 3 months, and then every 3 months. For most patients, follow-up evaluations at each visit included a physical examination, complete blood count, liver function test, serum CA 19–9 measurement, chest radiography, and dynamic CT scan of the abdomen and pelvis. Recurrence was proven by biopsy or cytology, and/or by radiological findings that indicated an increase in tumor size.

A pretreatment serum CA 19–9 of 400 U/mL, the significant cutoff point in our previous reports [13,14], or a 40% decrease in CA 19–9, close to the median value for each group, was chosen as a cutoff point for comparison of patient survival rates. Parameters in the capecitabine and 5-FU groups were compared using chi-square tests, Fisher’s exact tests, and t-tests, as applicable. Overall survival (OS) was defined as the interval from the commencement of CRT to the date of death or last follow-up and its probability was calculated by the Kaplan–Meier method. Univariate and multivariate analyses were performed using the log rank test and Cox’s proportional hazard model, respectively, to evaluate prognostic factors associated with OS. All statistical tests were two-sided and were performed using STATA software (ver. 9.0; Stata Corp., College Station, TX). *P* values less than 0.05 were considered statistically significant.

## Results

### Patient characteristics

The clinical parameters of the capecitabine and 5-FU groups were similar (Table 2). The median follow-up time for all patients was 12.3 months (range, 2.3–65.8 months) and was similar in the capecitabine and 5-FU groups [12.6 months (range, 2.3–39.2 months) vs. 11.2 months (range, 4.6–65.8 months), *p* =0.837].

**Table 2:** Comparison of toxicities^* ^**between the capecitabine and 5-fluorouracil (5-FU) groups**

	**Capecitabine (n = 52), n**				**5-FU (n = 46), n**				
**Type of toxic effect**	**Grade 1**	**Grade 2**	**Grade 3**	**Grade 4**	**Grade 1**	**Grade 2**	**Grade 3**	**Grade 4**	***p-value†***
Hematologic toxicity									
Leukopenia	22	8	0	0	6	9	1	1	0.007
Anemia	36	1	0	0	23	7	2	0	0.022
Thrombocytopenia	18	3	0	0	15	1	0	0	0.719
Nonhematologic toxicity									
Hand-foot syndrome	3	0	0	0	1	0	0	0	0.620
Anorexia	19	0	0	0	18	2	1	0	0.283
Vomiting	6	6	0	0	14	8	2	0	0.010
Diarrhea	5	2	0	0	6	2	1	0	0.860
Constipation	1	0	0	0	3	0	0	0	0.339
Abdominal pain	14	1	0	0	12	1	0	0	1.000
Stomatitis	21	0	0	0	30	0	0	0	0.016

Abbreviations: same as Table 1.

Some patients experienced more than one toxicity.

^*^National Cancer Institute Common Terminology Criteria for Adverse Events, version 3.0.

^†^ Fisher’s exact test, two-tailed.

### Toxicity

Treatment was well tolerated in both groups and there were no treatment-related deaths. The details of the distribution of toxicities between the capecitabine and 5-FU groups are summarized in Table 2. None of the patients in the capecitabine group developed Grade ≥3 toxicities, whereas 4 patients each (8.7%) in the 5-FU group developed Grade ≥3 hematologic and non-hematologic toxicities (*p* = 0.045 each).

### Tumor response and overall survival

Overall and primary tumor responses were evaluated one month after CRT by imaging modalities, except in one patient who had no available radiologic images after CRT. No patient in either group achieved a CR in primary tumor or overall response. Primary tumor response was as follows: PR in 29 patients (29.9%), SD in 67 (69.1%), and PD in 1(1.0%). Overall tumor response was as follows: PR in 14 patients (14.4%), SD in 25 (25.8%), and PD in 58 (59.8%). Of the 58 patients with overall tumor responses of PD, 57 (83.6%) had distant metastases, regardless of primary tumor response (i.e., 15 had primary tumor responses of PR and 42 of SD); one patient (1%) showed primary tumor progression without distant metastasis. Overall and primary tumor responses and percent decreases in CA 19–9 concentrations in the capecitabine and 5-FU groups are summarized in Table 3. None of these between group differences was statistically significant (*p* > 0.05 each). Of the 98 patients, 7 (7.1%) underwent surgical resection, with 5 being margin negative and 2 margin positive. Rates of conversion to resectability were similar in the capecitabine and 5-FU groups [4/52 (7.7%) vs. 3/46 (6.5%), *p* = 1.000]. Of the 7 resected patients, 2 developed locoregional recurrence, 3.5 and 5.5 months later, with the remaining 5 continuing to be locoregionally controlled. After completion of CRT, 47 patients (48%), received gemcitabine based chemotherapy until disease progression, treatment-limiting toxicity, or death; the median number of chemotherapy cycles was 3 (range, 1–17). The remaining 51 patients (52%) did not receive maintenance chemotherapy because of patient refusal or poor performance status. The distribution of patients receiving maintenance chemotherapy were similar in the capecitabine and 5-FU groups [26/52 (50%) vs. 21/46 (45.7%), *p* = 0.667].

**Table 3:** Tumor responses^* ^**in the capecitabine and 5-fluorouracil (5-FU) groups**

**Response**	**Capecitabine (n = 51**^**†**^**), n (%)**	**5-FU (n = 46), n (%)**	***p-*value**
Primary tumor			0.658^‡^
Complete response	0 (0)	0 (0)	
Partial response	16 (30.7)	13(28.2)	
Stable disease	35 (67.3)	32 (69.6)	
Progressive disease	0 (0)	1 (2.2)	
Overall			0.273^‡^
Complete response	0 (0)	0 (0)	
Partial response	7 (13.7)	7 (15.2)	
Stable disease	10 (19.6)	15 (32.6)	
Progressive disease	34 (66.7)	24 (52.2)	
CA 19–9 percent decrease (%)			
Median (range)	38.9 (-624.1–98.9)	39.7 (-383.4–98.6)	0.484^§^
Mean ± SE	-1.9 ± 19.5	16.2 ± 15.8	
<40	29 (55.8)	26 (56.5)	1.000^‡^
≥40	23 (44.2)	20 (43.5)	

^*^Response assessment in solid tumors (RECIST) version 1.1.

^†^No available radiologic image for one patient.

^‡^Fisher’s exact test, two-tailed.

^§^*t*-test, two-tailed.

**Figure 1:**
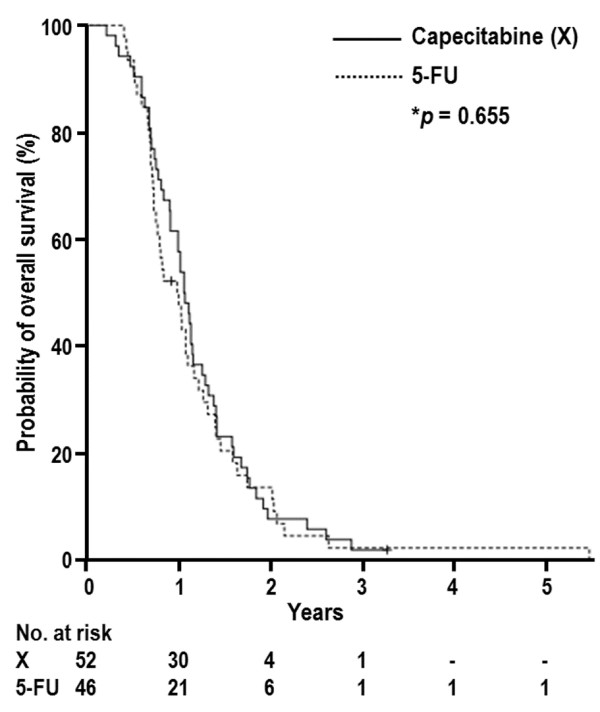
Overall survival curves of the capecitabine and 5-FU groups.^*^log-rank test.

At the time of analysis, 96 patients (92.9%) had died and 2 (7.1%) remained alive. The median OS time for all patients was 12.1 months [95% confidence interval (CI), 9.8–13.4 months] and the median OS times for the capecitabine and 5-FU groups were 12.5 months (95% CI, 10.7–13.7 months)and 11.6 months (95% CI, 8.5–13.8 months), respectively (*p* = 0.655) (Figure 1). Table 4 summarizes the results of univariate analysis for the prognostic factors associated with OS. Maintenance chemotherapy (No vs. Yes) was significantly associated with OS (10.6 months vs. 14.4 months, *p* < 0.001) and N classification (N0 vs. N1; 13.0 months vs. 10.7 months, *p* = 0.069) and pretreatment CA 19–9 level (< 400vs. ≥ 400 U/mL; 12.7 months vs. 9.7 months, *p* = 0.063) showed marginal associations with OS, whereas none of the other parameters was significantly associated with OS (*p* > 0.05) (Table 4). Multivariate analysis showed that pretreatment CA 19–9 level and maintenance chemotherapy were independent prognostic factors for OS (*p* < 0.05) (Table 5).

**Table 4:** Univariate analysis of factors influencing overall survival

		**Overall survival**	
		**Median (95% CI), months**	*p-*value^*^
Gender	Male	11.6 (9.2–14.0)	0.426
	Female	13.4 (12.2–14.6)	
Age (years)	<65	12.0 (10.9–13.1)	0.608
	≥65	12.1 (9.8–14.4)	
Tumor location	Head	11.6 (9.0–13.0)	0.413
	Body/tail	13.0 (9.5–15.6)	
Tumor size(cm)	<4	12.7 (9.6–13.8)	0.664
	≥4	12.0 (9.3–13.4)	
Histological differentiation	Well/moderate	13.4 (10.6–15.6)	0.717
	Poor	8.8 (5.9–25.7)	
	Not specified	12.2 (9.5–13.2)	
cN classification	N0	13.0 (10.6–15.0)	0.069
	N1	10.7 (8.8–12.7)	
CEA (ng/ mL)	<5	12.6 (9.8–13.4)	0.190
	≥5	11.8 (8.3–13.7)	
Pretreatment CA 19–9 level (U/mL)	<400	12.7 (11.6–15.0)	0.063
	≥400	9.7 (8.3–12.7)	
Concurrent chemotherapy	Capecitabine	12.5 (10.7–13.7)	0.655
	5-FU	11.6 (8.5–13.8)	
Primary tumor response	Responder	13.6 (8.6–18.9)	0.165
	Nonresponder	11.9 (9.6–13.0)	
CA 19–9 percent decrease (%)	<40	11.6 (9.0–13.1)	0.639
	≥40	12.7 (10.7–14.4)	
Post-CRT surgery	No	12.2 (9.8–13.4)	0.883
	Yes	12.0 (5.9–25.7)	
Maintenance chemotherapy	No	10.6 (8.5–12.1)	<0.001
	Yes	14.4 (11.7–18.8)	

Abbreviations: *Responder* complete or partial response, *Nonresponder* stable disease or progressive disease *NS* not significant, *p* > 0.05, others are same as in Table 1.

^*^Log rank test.

**Table 5:** Multivariate analysis of factors influencing overall survival

**Factor**		**Hazard ratio**	**95% CI**	*p*-value^*^
CA 19–9 level (U/mL)	<400	1.000		<0.001
	≥400	3.750	1.757–8.005	
Maintenance chemotherapy	No	1.000		<0.001
	Yes	0.218	0.096–0.495	

Abbreviations: *CI* confidence interval, other abbreviations are as in Table 1.

^*^Multivariate analysis using the Cox proportional hazards model.

## Discussion

We found that the rates of Grade ≥3 hematologic (0% vs. 8.7%, *p* = 0.045) and non-hematologic (0% vs. 8.7%, *p* = 0.045) toxicities were significantly lower in the capecitabine group than in the 5-FU group. Lower toxicity rates may have been due to the pharmacodynamic advantages of oral capecitabine relative to bolus 5-FU [15]. Capecitabine is a tumor-selective fluoropyrimidine carbamate that is converted to active 5-FU by TP, an enzyme of higher abundance in tumor than in normal tissue that is upregulated by radiation in tumor but not in normal tissue [9,10]. Thus, theoretically, capecitabine could show lower rates of toxicity than bolus 5-FU. A previous study found that the rate of Grade ≥3 hematologic toxicity was lower in patients receiving capecitabine than 5-FU during preoperative CRT for locally advanced rectal cancer (1.5% vs. 7.8%, *p* = 0.04), similar to our results, but that rates of Grade ≥3 diarrhea (8.6% vs. 2.1%, *p* = 0.006) and hand-foot syndrome (2%vs. 0%, *p* = 0.061) were higher in the capecitabine group [16]. However, a phase II trial of induction chemotherapy with gemcitabine and cisplatin followed by CRT with capecitabine in patients with locally advanced pancreatic cancer showed that the rates of Grade ≥3 diarrhea (5.4%) and hand-foot syndrome (0%) were low during CRT [17], similar to our findings. These findings implied that the lower rates of Grade ≥3 non-hematologic toxicity in the capecitabine than in the 5-FU group in our study maybe due to genetic differences in tolerability or susceptibility to capecitabine between Caucasians and Asians, as the conversion rate of tegafur to fluorouracil is different between Caucasians and Asians due to polymorphic differences in the CYP2A6 gene [18,19]. However, due to the relatively small number of patients in the capecitabine group (n = 52), which may be insufficient to determine the overall actual toxicity rates thoroughly and low incidence of toxicities in western study treated with CRT with capecitabine [20], more comprehensive and larger-scale studies should be needed.

Protracted infusion of 5-FU and capecitabine, which prolongs the exposure of non-cycling tumor cells to 5-FU, may enhance cytotoxicity relative to bolus 5-FU. Protracted infusion of 5-FU or capecitabine during adjuvant CRT has been shown to improve relapse-free survival (RFS) and OS, compared with bolus 5-FU, in patients with rectal cancer [16,21]. In contrast, the Intergroup 0144 study, which compared different 5-FU based chemotherapeutic regimens in rectal cancer, found that protracted and bolus infusion of 5-FU yielded similar RFS and OS, and our previous report [22] showed that capecitabine and bolus 5-FU resulted in similar radiologic and pathologic tumor responses in patients receiving preoperative CRT for rectal cancer. However, to date, it has been remained unclear whether chemotherapeutic regimens, among a protracted infusion of 5-FU, capecitabine, or bolus injection of 5-FU, can result in superior outcomes in patients with pancreatic cancer. We found that capecitabine, which mimics the protracted infusion of 5-FU, and bolus 5-FU yielded similar tumor responses and overall survival in patients with locally advanced pancreatic cancer.

A randomized trial found that, compared to5-FU, gemcitabine yielded better outcomes, including alleviation of disease-related symptoms and longer OS, in patients with advanced, symptomatic pancreatic cancer [23], and thus various dosages and schedules of gemcitabine based chemotherapy, with or without RT, have been tried to improve survival in locally advanced pancreatic cancer patients [4-6,8]. These studies, however, showed that gemcitabine based chemotherapy, with or without oxaliplatin, paclitaxel, docetaxel, and tyrosine kinase inhibitors, resulted in high rates of severe toxicities, without survival benefits, compared to 5-FU. Thus, 5-FU continues to be used as a concurrent chemotherapeutic agent during CRT [5]. To evaluate the effectiveness and safety of capecitabine, we compared outcomes, including toxicity, tumor response and overall survival, of RT plus capecitabine or bolus 5-FU, in patients with locally advanced pancreatic cancer.

This study was retrospective and thus had certain inherent limitations. First, it included relatively small numbers of patients in the capecitabine (n = 52) and 5-FU (n = 46) groups, which may have been insufficient to compare outcomes thoroughly. Second, this was a retrospective comparison study of two groups with different chemotherapeutic regimens, not a randomized trial. Thus, further larger scaled and comprehensive studies are required to accurately compare the outcomes of these two chemotherapy regimens in patients with locally advanced pancreatic cancer. However, in present study, the characteristics of patients in the two groups did not differ significantly, and each chemotherapeutic regimen was decided according to patient’s preferences.

## Conclusion

In conclusion, we compared the outcomes of CRT with capecitabine or bolus 5-FU in patients with locally advanced pancreatic cancer. We found that CRT with capecitabine had low toxicity rates, but yielded similar tumor responses and overall survival compared with CRT with bolus 5-FU. These findings suggest that capecitabine may be a safe and feasible chemotherapy regimen in patients with locally advanced pancreatic cancer treated with CRT.

## Competing interests

None of the authors have potential conflicts of interest.

## Authors’ contributions

THK, SMW, WJL and YJK are responsible for the study design. THK, WJL, SMW, YJK, TSK, SSH, BHK, SHM, SSK, YHK, SJP, DYK and JWP collected the clinical data and drafted the manuscript. THK, SMW, WJL, JK, DYK and YJK revised the manuscript. YJK, WJL and SMY collected the pathologic data and analysis THK, DYK, WJL and SMW were responsible for the treatment and evaluation of the patients. SJP, SHM, SSK, DYK, JWP and THK provided oversight of the analysis of data and reviewing of the manuscript. All authors read and approved the final manuscript.
